# PD209 Social Burden Of Inflammatory Bowel Diseases In Poland: Impact Of Changes In Drug Programs On Social Security Expenditures

**DOI:** 10.1017/S0266462324004318

**Published:** 2025-01-07

**Authors:** Grzegorz Binowski, Dominik Strządała, Karolina Dziadek, Agnieszka Nadzieja-Kozioł, Monika Jakubowska, Cezary Pruszko

## Abstract

**Introduction:**

Crohn’s disease and ulcerative colitis are chronic conditions that primarily affect young individuals of working age and can lead to severe and irreversible complications, which may result in the temporary or permanent inability to work. The objective of this report was to determine whether including broader reimbursement of innovative drugs would result in tangible benefits from a public expenditure perspective.

**Methods:**

Social security and social insurance fund benefits paid to incapacitated patients between 2012 and 2021 were used to measure the effectiveness of the innovative drug reimbursement policy. The analysis included expenditures per patient and the full population. Disability and sickness benefits were classified as public transfers and were not included in the costs for the pharmacoeconomic analyses. However, they are crucial in evaluating the efficacy of treatment for a specific disease because they broaden the perspective of public spending. An increase in social benefit expenses per patient suggests a decline in the patient’s health status, whereas a decrease indicates an improvement.

**Results:**

The cost of disability benefits for patients diagnosed with Crohn’s disease and ulcerative colitis was approximately EUR5,814 annually. However, the cost of reimbursing drugs used in drug programs per patient was notably lower. The analysis results indicated positive changes in the amount and structure of social benefits for this patient population (e.g., fewer patients receiving disability benefits). This conclusion was further strengthened by an analysis of the data for each province separately.

**Conclusions:**

The expansion of reimbursement for innovative drugs in the treatment of inflammatory bowel disease provides benefits for public spending.

